# Apparent temperature and acute myocardial infarction hospital admissions in Copenhagen, Denmark: a case-crossover study

**DOI:** 10.1186/1476-069X-11-19

**Published:** 2012-03-30

**Authors:** Janine Wichmann, Matthias Ketzel, Thomas Ellermann, Steffen Loft

**Affiliations:** 1 Section of Environmental Health, Department of Public Health, Faculty of Health Sciences, University of Copenhagen, Copenhagen, Denmark; 2 Department of Environmental Science, Aarhus University, Roskilde, Denmark; 3 Section of Environmental Health, Department of Public Health, University of Copenhagen, Øster Farimagsgade 5A, 1014 Copenhagen K, Denmark

**Keywords:** Temperature, Acute myocardial infarction, Hospital admissions, Case-crossover design

## Abstract

**Background:**

The influence of temperature on acute myocardial infarction (AMI) has not been investigated as extensively as the effects of broader outcomes of morbidity and mortality. Sixteen studies reported inconsistent results and two considered confounding by air pollution. We addressed some of the methodological limitations of the previous studies in this study.

**Methods:**

This is the first study of the association between the daily 3-hour maximum apparent temperature (Tapp_max_) and AMI hospital admissions in Copenhagen. The study period covered 1 January 1999-31 December 2006, stratified in warm (April - September) and cold (October - March) periods. A case-crossover epidemiology study design was applied. Models were adjusted for public holidays and influenza, confounding by PM_10_, NO_2 _and CO was investigated, the lag and non-linear effects of Tapp_max _was examined, effect modification by age, sex and SES was explored, and the results of the case-crossover models were compared to those of the generalised additive Poisson time-series and generalised estimating equation models.

**Results:**

14 456 AMI hospital admissions (12 995 people) occurred during the study period. For an inter-quartile range (6 or 7°C) increase in the 5-day cumulative average of Tapp_max_, a 4% (95% CI:-2%; 10%) and 9% (95% CI: 3%; 14%) decrease in the AMI admission rate was observed in the warm and cold periods, respectively. The 19-65 year old group, men and highest SES group seemed to be more susceptible in the cold period.

**Conclusion:**

An increase in Tapp_max _is associated with a decrease in AMI admissions during the colder months.

## Background

The influence of certain weather types (heat waves and air mass types), specific weather parameters, and also of the atmospheric environment in general, on human health, particularly all-cause mortality, has been studied extensively [1-4]. It is likely that the overall effect of temperature strongly depends on the general climate of the area, cause and type of health outcome (death or hospital admission), population characteristics (age, sex, socio-economic status (SES)), and the efficiency of the health system. Some of the effects of temperature may occur through pathways involving air pollution, but the effects of temperature on health, independent of air pollution, is also of interest.

The influence of temperature on morbidity and mortality from acute myocardial infarction (AMI) specifically has not been investigated as extensively as the effects of broader outcomes. In total 16 studies investigated the acute effects of temperature (various parameters) on AMI hospital admissions [5-11]. Bhaskaran et al summarised the evidence of 10 of the 16 studies [5]. Five and three of these 16 studies reported detrimental effects of cold and heat, respectively. A worldwide study (17 countries) and two from Italy and Korea reported statistically significant linear, weak and inverse associations between temperature parameters (all year) and AMI [6,7,11]. Three large studies not included in the review by Bhaskaran et al failed to detect an association between temperature and AMI [8-10].

Bhaskaran et al pointed out that further research with consistent methodology is required to clarify the magnitude of these effects and to identify susceptible groups [5]. Thirteen of the 16 studies applied generalised additive Poisson time-series models (GAM) [5-7,10], one applied negative binomial models [11], one applied generalised linear Poisson models [9] and one the case-crossover design [8]. Six of the 16 studies allowed for non-linear temperature effects [5,8,10] and seven studies investigated lag effects [5,8-10].

Bhaskaran et al also summarised the evidence from 16 time-series and case-crossover studies that investigated the acute effects of air pollution on AMI [12]. The evidence suggests that ambient air pollution exposure, especially PM_2.5 _(particulate matter with an aerodynamic diameter less than 2.5 μm), is detrimental to AMI hospital admission risk. Yet, only two of the 16 studies that focused on the association between temperature and AMI considered also confounding by air pollution, specifically PM_2.5 _(particulate matter with an aerodynamic diameter less 2.5 μm in diameter, PM_10_, ozone (O_3_), nitrogen dioxide (NO_2_) and carbon monoxide (CO) and sulphur dioxide (SO_2_) [5,8].

This is the first study on the association between the daily 3-hour maximum apparent temperature (Tapp_max_) and AMI hospital admissions (> 18 years) in Copenhagen during an 8-year study period (1999-2006). We attempted to address some of the limitations of the previous studies: adjusted models for public holidays and influenza, investigated confounding by ambient air pollution (PM_10_, NO_2 _and CO), examined the lag and non-linear effects of Tapp_max_, explored effect modification by age, sex and SES, and compared the results of the case-crossover models to those of the GAM and generalised estimating equation (GEE) analyses.

## Materials and methods

### Hospital admission data

Hospital admission data were retrieved from the Danish Hospital Register for inhabitants of Copenhagen (postal code < 2930, ≤ 15 km radius from the city centre, population ≈ 1 million) who were > 18 years and lived in the area between 1 January 1999-31 December 2006. Acute myocardial infarction (AMI) hospital admissions, with a primary diagnosis coded I21 - I22 according to the International Classification of Diseases 10th Revision (ICD 10), were included as a health outcome. With duplicate hospital admissions (mostly due to an emergency room admission) on the same day, the observation with the longest stay in hospital was retained.

AMI hospital admissions that occurred within 28 days after a previous AMI hospital admission were excluded (2585 admissions) as readmissions following discharge for AMI are quite high [13].

### Meteorological and air pollution data

Meteorological and air pollution data were collected at a fixed single urban background monitor and provided by the Department of Environmental Science, Aarhus University [14]. Air pollution data included measurements of PM_10 _(Beta attenuation by SM200 monitor; Opsis, Sweden), NO_2 _(M 200A; API, San Diego, USA) and CO (M 300 monitor; API, San Diego, USA). PM_10_, NO_2 _and CO were modelled as 24-hour averages (midnight to midnight).

PM_2.5 _and O_3 _were not considered as confounders due to the large number of days with missing values. SO_2 _levels are extremely low in Copenhagen and the pollutant is not monitored anymore. Meteorological data included measurements of temperature and relative humidity (HMP45A, Vaisala, Finland).

We used apparent temperature as exposure variable because this is a construct intended to reflect the physiological experience of combined exposure to humidity and temperature and thereby better capture the response on health than temperature alone [1,2,15]. The daily 3-hour maximum apparent temperature (Tapp_max_) was selected as the primary exposure variable in order to compare our results to that of a large European study conducted in 12 cities and to a study from Copenhagen [15,16]. Barnett et al concluded in a review that there is no single temperature measure that is superior to others [17]. Tapp_max _is defined as the highest value of the eight 3-hourly apparent temperature averages on a specific day. The eight 3-hourly apparent temperature averages were calculated from midnight-3 am, 3 am-6 am, 6 am-9 am and so forth to 9 pm-midnight.

(1) $$\begin{array}{c} \text{Saturation}\;\text{vapour}\;\text{pressure}\\ \text{ = }6.112^{\prime }10^{\left(7.5\times \;\text{temparature}{\;}^{\circ }\text{C/}(273.7\text{ + temparature}{\;}^{\circ }\text{C}\right)} \end{array}$$

(2) $$\begin{array}{c} \text{Actual vapour pressure}\\ =\;\left(\text{relative humidity }\left(\%\right)\times \text{saturation vapour pressure}\right)/\text{1}00 \end{array}$$

(3) $$\begin{array}{c} {\text{Dew point temperature }}^{\circ }\text{C}\\ =\;\left(-\text{43}0.\text{22 }+\text{ 237}.\text{7}\times \text{ln}\left(\text{actual vapour pressure}\right)\right)/\left(-\text{ln}\left(\text{actual vapour pressure}\right)\;+\text{ 19}.0\text{8}\right) \end{array}$$

(4) $$\begin{array}{c} {\text{Apparent temperature }}^{\circ }\text{C}\\ =-\text{2}.\text{653 }+\;\left(0.\text{994}\times {\text{temperature }}^{\circ }\text{C}\right)\;+\;0.0\text{153}\times \left({\text{dew point temperature }}^{\circ }\text{C}\right) \end{array}$$

The measurements of relative humidity have a minor error, which is most likely due to the calibration. However, this has only a minor impact on the calculation of Tapp_max _(Equations 1-4, Additional file 1. Figures S1 and S2) and is expected not to reduce the validity of the results from this study. The relative humidity data were applied in two other studies in Copenhagen [16,18]. During the study period there were 569 and 114 days with missing values for the pollutants and meteorological variables, respectively, with a total of 625 days with missing data out of 2 922 days. Missing data were excluded from the regression models.

### Influenza data

Influenza epidemics data were provided by the National Serum Institute as weekly percentage of total general physician's consultations due to influenza in Denmark, whereas city level data were not available.

### Socio-economic status data

Addresses of the 12 995 hospitalised persons were retrieved by linkage with the Danish Central Population Registry. A report was published on SES groups in Greater Copenhagen, which classified communities and the inner city neighbourhoods into four SES groups (highest, second highest, second lowest and lowest), based on household income, educational and employment status [19]. The majority (92%) of the hospitalised persons lived at one address during 1999 to 2006. A SES class could not be assigned to 1 002 cases (739 people) due to invalid street codes. A SES code was assigned for the valid address at which the person lived longest. In the case of more than three addresses, the mode of the area SES classes at the different addresses was assigned to that person.

### Ethics

As this study was purely registry based, no human participants were recruited or included in experiments. Approval was granted by the proper authority, which is the Danish Data Protection Agency.

### Statistical analysis

To investigate the association between Tapp_max _and AMI hospital admissions we used the case-crossover design which was developed as a variant of the case-control design to study the effects of transient exposures on acute events, comparing each person's exposure in a time period just prior to a case-defining event with person's exposure at other times [20]. The time-stratified case-crossover design was applied by defining the day of admission as the case day and same day of the week in the same month and year as control days [20]. Hereby, control on all measured and unmeasured personal characteristics that do not vary over time is accomplished. If in addition, the control days are chosen close to the event day, personal characteristics that vary slowly over time are also controlled by matching. With this approach even very strong confounding of exposure by seasonal patterns is controlled by design [21,22].

The data were analysed using conditional logistic regression analysis (PROC PHREG in SAS 9.2, SAS Institute, Cary, NC). Public holidays were controlled for by use of a binary variable and influenza as a linear variable. Previous studies in Copenhagen reported a linear relationship between air pollutants and cardiovascular admissions [18,23]. PM_10_, NO_2 _and CO were therefore modelled as linear terms, one pollutant at a time.

Lag0 (same day exposure as day of admission) to lag5 (exposure five days prior to day of admission) of Tapp_max _were investigated, as well as cumulative averages: mean of lag0-1 (1-day cumulative average, CA2), and up to mean lag0-4 (CA5). Control days for lag1 to lag5 were defined as for lag0. The values of the cumulative averages were set as missing if any of the values needed for computing them were missing.

There is no standard method to select lags. Most studies select a lag that is significant and has the lowest Akaike Information Criterion (AIC) [24]. The lag of Tapp_max _with the lowest AIC was applied in the stratified models. In general, the lowest AIC model had the strongest association between Tapp_max _and AMI admissions.

Hazard ratios (HR) and the 95% confidence intervals (CI) were calculated per inter-quartile range (IQR) increase in Tapp_max _(in°C). The results are presented as the percent excess risk in AMI admissions per IQR increase in Tapp_max _using the following calculation: [(exp^β×IQR^) - 1] × 100%, where β is the estimated coefficient in the model.

Models were first stratified by seasonal period (warm or cold) and then by sex, age groups and SES. The warm and cold periods were defined as April-September and October-March, respectively, as in other studies [1-3,16,18]. Age was categorised as 19-65, 66-80 and > 80 years.

Several sensitivity analyses were applied. The linearity and strength of the association between Tapp_max _and AMI admissions were checked in GAM models with the use of the *gam *procedure, *mgcv *package in R statistical software (R Development Core Team, 2010). Models were adjusted for day of the week (as dummy variables), public holidays (as a binary variable) and influenza (as a linear variable). Models were run with linear and non-linear terms for Tapp_max_, as a smoothing spline function with 4 degrees of freedom (df). A spline function, defined by piecewise polynomials, has a flexible shape that is useful for adjusting for non-linear effects. The smoothness of a spline is a function of the number of degrees of freedom. We investigated whether the non-linear term for Tapp_max _improved the models by conducting log-likelihood ratio tests. Unmeasured, unknown and potentially variable seasonal and long term patterns need to be controlled for adequately in GAM models, whilst still leaving sufficient information from which to estimate temperature effects. Smoothing splines of calendar time with 3 df/year were used in the cold and warm periods to control for long-term trend and seasonality. Models with a range of alternative df for calendar time were run and the Tapp_max _estimates were robust.

Another sensitivity analysis was to apply GEE models, as done in a large European study [15,25]. The observations among seasonal periods of different years were assumed to be independent, whereas daily counts of hospital admissions within each period were considered to be correlated. A first-order autoregressive structure was applied and takes into account the intra-period correlation, where observations close in time tend to be more correlated than distant observations. A Poisson distribution of the outcome variable was assumed. We repeated our analysis with such a GEE approach (PROC GENMOD in SAS 9.2, SAS Institute, Cary, NC). Models were adjusted for day of the week (as dummy variables), public holidays (as a binary variable) and influenza (as a linear variable).

## Results

Table 1 displays a summary of the meteorological conditions, air pollution levels and influenza epidemics during the study period. None of the EU air quality limit values (PM_10 _40 μg.m^-3 ^(annual), NO_2 _21 ppb (annual), CO 5.3 ppm (1-hour max)) were exceeded at the urban background monitoring site whereas street levels of PM_10 _and NO_2 _showed some exceedance (not shown) [14]. Table 2 displays the Spearman correlations between Tapp_max _and air pollutants in the warm and cold periods.

**Table 1:** Air pollutant levels and meteorological conditions^a ^in Copenhagen and weekly influenza visits in Denmark during study period (1 January 1999-31 December 2006)

	All year	Warm period	Cold period
Number of days	2922	1464	1458
Tapp_max _(°C)			
Number of days with missing data	114	32	82
Mean ± SD	10 ± 8	16 ± 6	4 ± 5
Minimum	-8	0	-8
Maximum	30	30	18
Percentiles			
25^th^	3	12	0
50^th^	9	16	3
75^th^	16	20	7
Inter-quartile range	13	8	7
PM_10 _(μg.m^-3^)			
Number of days with missing data	454	266	188
Mean ± SD	27 ± 16	27 ± 14	28 ± 17
Inter-quartile range	14	13	16
NO_2 _(ppb)			
Number of days with missing data	164	109	55
Mean ± SD	12 ± 5	11 ± 4	13 ± 5
Inter-quartile range	7	6	7
CO (ppm)			
Number of days with missing data	129	81	48
Mean ± SD	0.28 ± 0.10	0.23 ± 0.07	0.33 ± 0.10
Inter-quartile range	0.120	0.081	0.123
Weekly GP visits due to influenza in Denmark (%)			
Number of weeks with missing data	0	0	0
Mean ± SD	1.12 ± 1.50	0.28 ± 0.61	1.96 ± 1.65

^a^For lag0. Warm period: April - September, Cold period: October - March. SD: Standard deviation, GP: General practitioner

**Table 2:** Spearman correlation coefficients between Tapp_max _and pollutants^a ^in Copenhagen during 1 January 1999-31 December 2006

Warm period	**PM**_**10**_	**NO**_**2**_	CO
Tapp_max_			
**Correlation**	0.324	0.094	-0.176
**Number of days^b^**	1172	1336	1366
**p-value**	< 0.0001	0.0006	< 0.0001

**PM_10_**			
**Correlation**	-	0.465	0.454
**Number of days^b^**	-	1169	1122
**p-value**	-	< 0.0001	< 0.0001

**NO_2_**			
**Correlation**	-	-	0.622
**Number of days^b^**	-	-	1295
**p-value**	-	-	< 0.0001

**Cold period**	**PM_10_**	**NO_2_**	**CO**

**Tapp_max_**			
**Correlation**	0.053	0.034	-0.252
**Number of days^b^**	1217	1353	1361
**p-value**	0.067	0.214	< 0.0001
**PM_10_**			
**Correlation**	-	0.457	0.530
**Number of days^b^**	-	1245	1250
**p-value**	-	< 0.0001	< 0.0001
**Cold period**	**PM_10_**	**NO_2_**	**CO**
**NO_2_**			
**Correlation**	-	-	0.723
**Number of days^b^**	-	-	1402
**p-value**	-	-	< 0.0001

^a^For lag0.

^b^Number of days is less than those in Table 1 due to missing data for Tapp_max_

and the pollutants.

Warm period: April - September, Cold period: October - March

Data for 2 922 days with 14 456 AMI hospital admissions (12 995 people) were available for analysis. The majority of the admissions were the first admission (90%) during the study period 1999-2006, with 8% and 1% being the second and third admission for an individual, respectively. Few of the admissions (0.4%) were from individuals who had four to seven admissions. Most of hospitalisations were for less than seven days (67%). Few of the admissions were fatal on the day of admission (309 deaths).

The pattern of admissions varied as expected by season, with more admissions during the cold period. The majority of the admissions (warm and cold periods combined) occurred in the oldest age group (> 80 years), men and lowest two SES (Table 3).

**Table 3:** Association between Tapp_max _(in°C) and acute myocardial infarction hospital admissions expressed as percentage increase in risk (%) and 95% confidence intervals per inter-quartile range increase in 5-day cumulative average of Tapp_max _(in°C) during the warm and cold periods of 1 January 1999-31 December 2006 in Copenhagen

	**Warm**^**a**^					**Cold**^**a**^				
	
	n^b^	IQR^c^	%	95% CI		n^b^	IQR^c^	%	95% CI	
**All**	6334	7	-4.4	-10.8	2.4	6750	6	**-8.9**	**-14.4**	**-3.0**
**Age categories**										
19 - 65 years	2017	7	-6.0	-16.7	6.0	2135	7	**-15.2**	**-25.6**	**-3.2**
66 - 80 years	2373	7	-1.2	-11.9	10.7	2445	6	-4.5	-13.9	5.8
> 80 years	1944	7	-6.7	-17.8	5.9	2170	6	-9.4	-19.0	1.3
**Sex**										
Women	2636	7	-7.5	-17.0	3.1	2760	6	-5.8	-14.6	4.0
Men	3698	7	-2.3	-10.7	6.9	3990	7	**-12.7**	**-20.6**	**-4.0**
**Socio-economic status**										
Lowest	1876	7	-10.0	-20.8	2.3	2013	6	-8.0	-18.1	3.3
Second lowest	1898	7	5.4	-7.2	19.7	2016	7	-4.1	-16.2	9.7
Second highest	1468	7	-9.0	-21.3	5.2	1508	7	-8.6	-21.5	6.5
Highest	722	7	-3.8	-21.5	17.8	797	6	**-25.1**	**-37.6**	**-10.1**

^a^Adjusted for public holidays and influenza rates, but not for any pollutants

^b^Number of admissions

^c^IQR different from Table 1, as values of lag0 and 5-day cumulative average of Tapp_max _differ. The IQR may also differ across groups as there was not an AMI hospital admission on each day for the different groups, hence the range of Tapp_max _may differ across groups.

**Figure 1:**
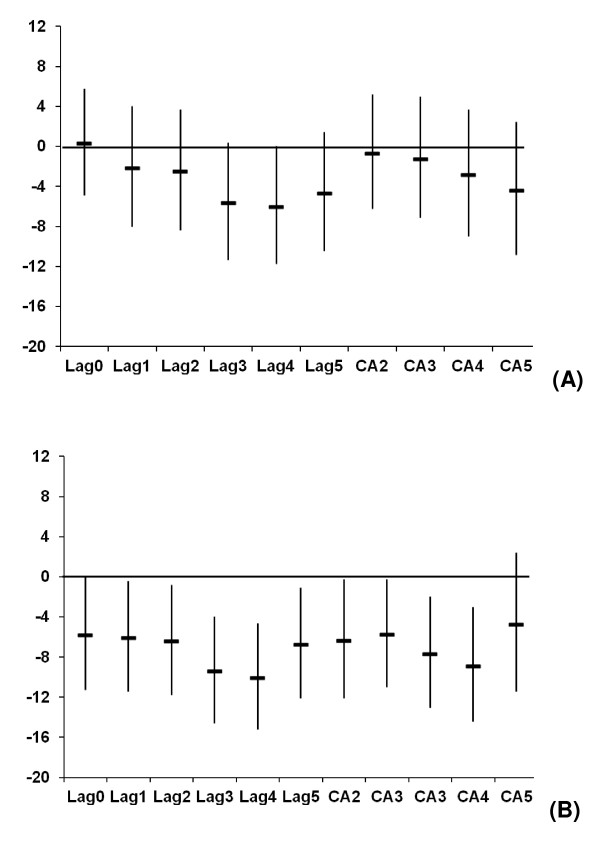
**Percentage change (95% CI) in AMI hospital admissions in Copenhagen per inter-quartile range increase in Tapp_max _during the (A) warm and (B) cold periods (1 January 1999-31 December 2006), adjusted for public holidays and influenza (not for any pollutants)**.

Figure 1 illustrates the % change in the AMI admissions per IQR increase in the different lags of Tapp_max _during the warm and cold periods, respectively, after adjusting for public holidays and weekly influenza rates, but not for any of the pollutants. In general the strongest association was observed between the CA5 of Tapp_max _and the AMI admissions (lowest AIC and/or statistically significant). The CA5 of Tapp_max _was selected to investigate susceptibility (Tables 3, 4, 5) and to compare the results from the case-crossover analysis with that of the GAM and GEE models (Table 6).

**Table 4:** Association between Tapp_max _(in°C) and acute myocardial infarction hospital admissions expressed as percentage increase in risk (%) and 95% confidence intervals per inter-quartile range increase in the 5-day cumulative average of Tapp_max _(in°C) during the warm period of 1 January 1999-31 December 2006 in Copenhagen

	**Adjusted for PM**_**10**_^**a**^					**Adjusted for NO**_**2**_^**a**^					**Adjusted for CO**^**a**^				
	**n^b^**	**IQR^c^**	**%^b^**	**95% CI**		**n^b^**	**IQR^c^**	**%^b^**	**95% CI**		**n^b^**	**IQR^c^**	**%^b^**	**95% CI**	

**All**	4939	7	-3.8	-12.6	5.9	5821	7	-7.0	-14.1	0.7	6002	7	-4.8	-11.5	2.4
**Age categories**															
19-65 years	1564	7	-3.5	-18.5	14.2	1851	7	-7.3	-19.3	6.5	1921	7	-6.8	-17.9	5.8
66-80 years	1840	7	-2.6	-16.8	14.0	2175	7	-3.8	-15.5	9.7	2245	7	0.9	-10.5	13.7
> 80 years	1535	7	-5.5	-20.7	12.7	1795	7	-10.6	-22.6	3.4	1836	7	-9.4	-20.7	3.6
**Sex**															
Women	2058	7	-6.9	-19.9	8.2	2414	7	-12.4	-22.6	-0.8	2481	7	-8.1	-18.0	3.0
Men	2881	7	-1.5	-13.1	11.7	3407	7	-3.0	-12.6	7.5	3521	7	-2.4	-11.2	7.3
**Socio-economic status**															
Lowest	1451	7	-11.8	-26.1	5.1	1725	7	-9.0	-21.4	5.4	1781	7	-9.8	-21.1	3.2
Second lowest	1499	7	3.6	-13.1	23.4	1744	7	-0.3	-13.8	15.3	1785	7	4.1	-8.9	19.0
Second highest	1140	7	-8.4	-25.4	12.5	1339	7	-13.4	-26.7	2.3	1394	7	-9.5	-22.3	5.4
Highest	546	7	10.8	-16.7	47.4	676	7	-3.8	-23.8	21.4	686	7	-4.6	-23.0	18.1

^a^Adjusted for public holidays and influenza rates in single pollutant models

^b^Number of admissions

^c^IQR different from Table 1, as values of lag0 and 5-day cumulative average of Tapp_max _differ. The IQR may also differ across groups as there was not an AMI hospital admission on each day for the different groups, hence the range of Tapp_max _may differ across groups.

**Table 5:** Association between Tapp_max _(in°C) and acute myocardial infarction hospital admissions expressed as percentage increase in risk (%) and 95% confidence intervals per inter-quartile range increase in the 5-day cumulative average of Tapp_max _(in°C) during the cold period of 1 January 1999-31 December 2006 in Copenhagen

	**Adjusted for PM**_**10**_^**a**^					**Adjusted for NO**_**2**_^**a**^					**Adjusted for CO**^**a**^				
	**n^b^**	**IQR^c^**	**%^b^**	**95% CI**		**n^b^**	**IQR^c^**	**%^b^**	**95% CI**		**n^b^**	**IQR^c^**	**%^b^**	**95% CI**	

**All**	5395	7	**-12.7**	**-20.0**	**-4.7**	6516	6	**-9.9**	**-15.6**	**-3.9**	6598	6	**-8.9**	**-14.4**	**-2.9**
**Age categories**															
19-65 years	1719	7	**-18.1**	**-30.2**	**-3.8**	2072	7	**-16.9**	**-27.4**	**-4.8**	2097	7	**-15.0**	**-25.5**	**-2.9**
66-80 years	1925	6	-6.1	-17.0	6.1	2356	6	-4.3	-13.9	6.3	2381	6	-4.2	-13.6	6.3
> 80 years	1751	7	-13.0	-25.4	1.4	2088	6	**-11.3**	**-20.9**	**-0.5**	2120	6	-9.8	-19.4	0.9
**Sex**															
Women	2195	6	-9.7	-19.6	1.5	2656	6	-4.8	-13.9	5.4	2690	6	-5.7	-14.6	4.2
Men	3200	7	**-13.7**	**-23.1**	**-3.3**	3860	7	**-15.3**	**-23.2**	**-6.6**	3908	7	**-12.5**	**-20.5**	**-3.7**
**Socio-economic status**															
Lowest	1598	6	-9.9	-21.7	3.7	1940	6	-6.8	-17.3	5.0	1968	6	-8.8	-18.9	2.6
Second lowest	1624	7	-3.2	-17.4	13.4	1938	7	-5.6	-17.8	8.3	1961	7	-3.2	-15.5	10.9
Second highest	1200	7	-14.4	-28.8	3.0	1469	7	-9.6	-22.7	5.9	1487	7	-7.8	-20.9	7.5
Highest	629	6	**-26.6**	**-41.0**	**-8.6**	767	6	**-27.1**	**-39.7**	**-12.0**	776	6	**-25.7**	**-38.2**	**-10.7**

^a^Adjusted for public holidays and influenza rates in single pollutant models

^b^Number of admissions

^c^IQR different from Table 1, as values of lag0 and 5-day cumulative average of Tapp_max _differ. The IQR may also differ across groups as there was not an AMI hospital admission on each day for the different groups, hence the range of Tapp_max _may differ across groups.

**Table 6:** Association between Tapp_max _(in°C) and acute myocardial infarction hospital admissions, expressed as percentage increase in risk (%) and 95% confidence intervals per inter-quartile range increase in 5-day cumulative average of Tapp_max _(in°C) during 1 January 1999-31 December 2006 in Copenhagen

	**Warm**^**a**^					**Cold**^**a**^				
	
	n^c^	IQR^d^	%	95% CI		n^c^	IQR^d^	%	95% CI	
GAM										
No pollutant	1360	7	-4.2	-9.9	1.9	1291	6	**-6.1**	**-11.0**	**-0.9**
PM_10_^b^	1049	7	-1.1	-9.0	7.4	1039	7	**-8.6**	**-15.1**	**-1.7**
NO_2_^b^	1243	7	-4.7	-11.1	2.1	1255	6	**-6.7**	**-11.7**	**-1.5**
CO^b^	1294	7	-5.4	-11.2	0.8	1267	6	**-6.0**	**-11.0**	**-0.8**
GEE										
No pollutant	1360	7	**-6.1**	**-10.4**	**-1.5**	1291	6	**-6.4**	**-10.3**	**-2.3**
PM_10_^b^	1049	7	**-6.2**	**-11.1**	**-0.9**	1039	7	**-9.3**	**-12.9**	**-5.6**
NO_2_^b^	1243	7	**-4.7**	**-8.9**	**-0.3**	1255	6	**-6.9**	**-10.8**	**-2.8**
CO^b^	1294	7	**-6.1**	**-10.2**	**-1.9**	1267	6	-4.9	-9.7	0.1

Warm period: April - September, Cold period: October - March

^a^Adjusted for day of the week, public holidays and influenza rates

^b^Single pollutant models adjusted for 5-day cumulative average of pollutant

^c^Number of days used in GAM and GEE model, which is less than those in Table 1 due to missing data for the 5-day cumulative average and Tapp_max _and the pollutant.

^d^IQR different from Table 1, as values of lag0 and 5-day cumulative average of Tapp_max _differ.

**Figure 2:**
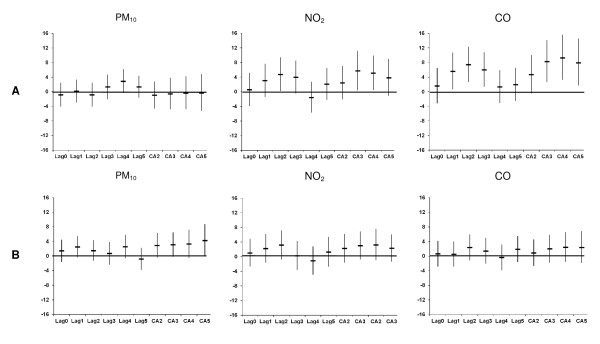
**Percentage change (95% CI) in AMI hospital admissions in Copenhagen per inter-quartile range increase in PM_10_, NO_2 _and CO during the (A) warm and (B) cold periods (1 January 1999-31 December 2006), adjusted for Tapp_max_, public holidays and influenza**.

Figures 2 illustrates the % change in the AMI admissions per IQR increase in the different lags of PM_10_, NO_2 _and CO during the warm and cold periods, respectively, after adjusting for Tapp_max _(same lag as pollutant), public holidays and weekly influenza rates. With respect to PM_10 _there were no sign of any association in the warm period and some borderline or insignificant positive associations in the cold period. For NO_2 _there were statistically significant and positive associations with AMI admissions in the warm period for CA3 and CA4. AMI hospital admissions showed significant and robust associations with CO levels in the warm period only and strongest with lag1 to lag3, and CA3 to CA5.

**Figure 3:**
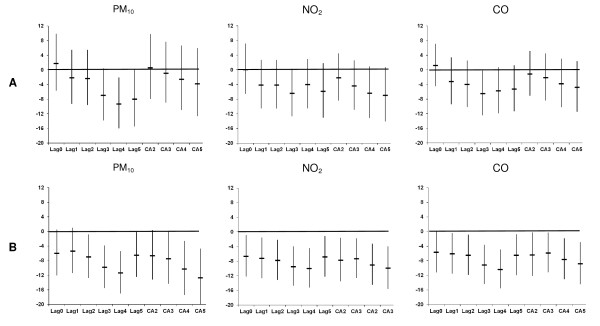
**Percentage change (95% CI) in AMI hospital admissions in Copenhagen per inter-quartile range increase in Tapp_max _during the (A) warm and (B) cold periods (1 January 1999-31 December 2006), adjusted for public holidays, influenza and PM_10_, NO_2 _or CO**.

Figure 3 illustrates the % change in the AMI admissions per IQR increase in the different lags of Tapp_max _during the warm and cold periods, respectively, after adjusting for the pollutants (same lag as Tapp_max_), public holidays and weekly influenza rates. In general the associations between Tapp_max _and AMI admissions were attenuated by the pollutants. The results in Tables 4 and 5 were thus reported after adjusting for the pollutants.

In the warm period an IQR increase in the CA5 of Tapp_max _was associated with an insignificant decrease of 4% (95% CI: 3%; 14%) in AMI admissions (Table 3), and after adjusting for PM_10_, NO_2 _and CO the decrease remained insignificant and was 4%, 7% and 5% respectively (Table 4). No susceptible groups were identified in the warm period (Tables 3 and 4).

In the cold period an IQR increase in the CA5 of Tapp_max _was associated with a significant decrease of 9% (95% CI: 3%; 14%) in AMI admissions (Table 3), and after adjusting for PM_10_, NO_2 _and CO the decrease remained significant and was 13%, 10% and 9% respectively (Table 5). Stronger associations were observed for the 19-65 year age group, men and the highest SES, with and without adjusting for the pollutants (Tables 3 and 5).

SES had a weak correlation with age (*r *= 0.13, p < 0.0001) and sex (*r *= 0.03, p < 0.0001). Age also had a weak correlation with sex (*r *= 0.21, p < 0.0001).

**Figure 4:**
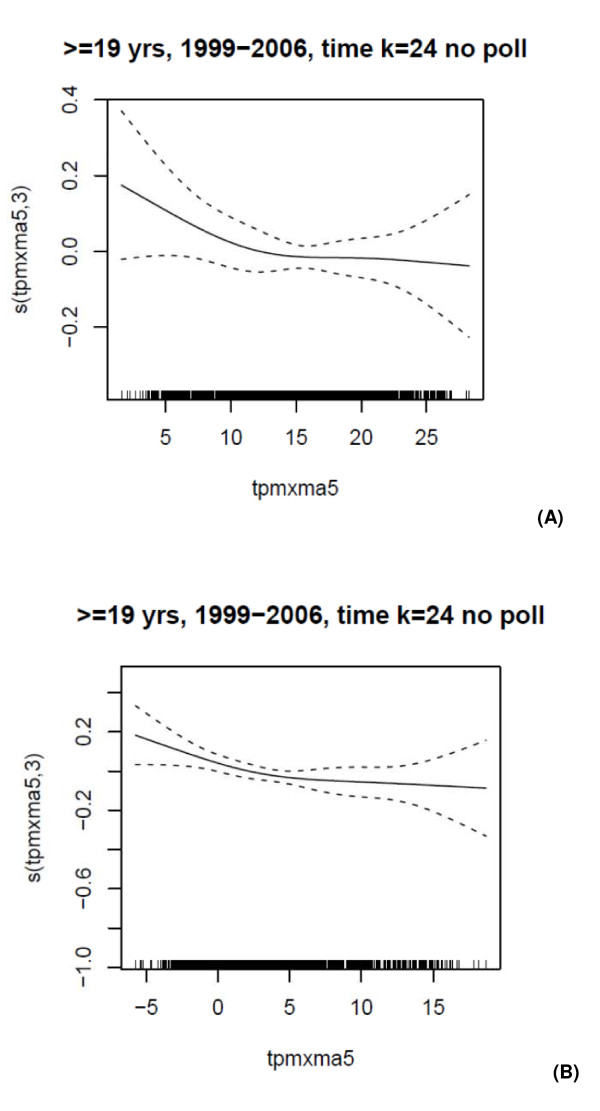
**Association between AMI hospital admissions in Copenhagen and 5-day cumulative average of Tapp_max _(per 1°C) during the (A) warm and (B) cold periods of 1 January 1999-31 December 2006, adjusted for day of the week, public holidays and influenza rates**.

The linearity of the association between the CA5 of Tapp_max _and AMI admissions was confirmed in the GAM analyses with and without adjusting for the pollutants (Figure 4). The GAM and GEE analyses (with and without adjusting for pollutants) confirmed the protective effect of an increase in Tapp_max _in the cold period, with somewhat weaker associations than those of the case-crossover analyses (Table 6). Although some of the associations were weaker or stronger than in the case-crossover analysis, all warm season associations were still insignificant in the GAM analysis. In the warm period the GEE analysis indicated that all associations were significantly protective and generally stronger than those of the case-crossover analyses.

## Discussion

This is the first study to evaluate the association between temperature and AMI hospital admissions in Copenhagen. We observed an apparent protective effect of high Tapp_max _on AMI admissions in the cold period of -1.5% per 1°C (95% CI: -2.6% - -0.5%), whereas the association was not statistically significant the warm period (-0.6% per 1°C (95% CI: -1.6% - 0.3%)).

Our results in the cold season are consistent with and comparable to five of the previous 16 studies on AMI and temperature, i.e. statistically significant short-term increased risk of AMI at lower temperatures [5]. None of these five studies investigated confounding by air pollution [5]. However, we found no adverse effects of high temperatures or protection during the warm season. This is in contrast to three of the previous 16 studies, particularly for temperature increases over a high range [5]. However, one of these three studies investigated confounding by air pollution [5]. Possibly, the temperatures reached in Copenhagen are not sufficient to cause adverse effect with respect to AMI. On the other hand three large studies failed to detect an association between temperature and AMI (all year) [8-10]. One of these three studies investigated confounding by air pollution [8].

Contrary to our present results on AMI, we have previously found a protective effect of high Tapp_max _on total CVD emergency admissions in Copenhagen during the warm period, but not in the cold period [16]. A large European study also reported weak protective effects of high Tapp_max _on total CVD admissions (emergency or planned) in North-Continental European cities in the warm period [15]. A study from California, USA reported slightly stronger effects of increasing daily mean temperature (Temp_mean_) on AMI than CVD hospital admissions (all year) [8].

A worldwide study (17 countries) and two from Italy and Korea reported statistically significant linear, weak and inverse associations between temperature (all year) and AMI [6,7,11], as in our study for the cold period. None of these three studies investigated confounding by air pollution.

Our lag structure with main apparent effects on AMI admissions occurring within a week is compatible with the patterns observed elsewhere [5-10]. Apart from the general lack of control for confounding by air pollution, other factors may explain the heterogeneity of risk estimates. These factors are related to the statistical methods used (different study designs, different lags selected, lack of control for confounding by day of the week, seasonality and influenza), the demographical profile of the study population (e.g. age, sex, SES), general climate of the study location, the efficiency and accessibility of the health system, diagnostic criteria of AMI, cause and type of CVD hospital admission.

In this study, the strongest associations were observed between Tapp_max _in the cold period and AMI admissions amongst men, the 19-65 year age group and the highest SES. Men in general have a higher risk to develop CVD than premenopausal women [26]. The 19-65 year group may be more susceptible due to more outdoor activities in the cold period compared to the elderly [3]. In Denmark, the highest SES group is in general more physically active and more likely to live in owned detached houses [27]. It has been found that snow shovelling may increase ischaemic heart disease [3]. Few of the previous 16 studies on AMI and temperature explored susceptibility to temperature effects according to individual-level characteristics [5-7,11]. Among those considering the effects of temperature separately for different age groups and sex, there were inconsistent results.

The mechanism by which cold ambient conditions can increase the risk of CVD remains unclear and more mechanistic research is needed [3,5]. However, there are several factors which have been shown to have clear seasonal variations, including plasma cholesterol, plasma fibrinogen, blood pressure, and red and white blood cell counts increases during winter [28].

Although the focus of this study is on Tapp_max_, the lack of an association between AMI hospital admissions with PM_10 _and NO_2 _does warrant some discussion. We found significant association between AMI admissions and CO levels in the warm period only. For an IQR (0.072 ppm) increase in the CA5 of CO (urban background levels), a 7% (95% CI: 1%; 13%) increase in the AMI admission rate was observed in the warm period. CO is not expected to be causative at these levels, but can be considered as an indicator of urban background exposure to air pollution from traffic and wood combustion in Copenhagen [18]. NO_2 _in the urban environment is mainly an indicator of air pollution from traffic and we found some associations with AMI admissions in the warm period, although not as robust as for CO. Outdoor levels of traffic pollutants might be more relevant in the warm period due to more outdoor activities or indoor penetration through open windows. Ischemic stroke, which is partly similar to AMI in pathogenesis, has previously been found to be significantly associated with urban background levels of CO and NO_x _(lag4) in Copenhagen, whereas the association with PM_10 _was less strong [29]. Bhaskaran et al concluded in a review that the evidence suggests that ambient air pollution exposure is detrimental to AMI hospital admission risk, with the risk increasing by 5-17% for each 10 μg.m^-3 ^increase in PM_2.5 _exposure [12]. This latter measure of exposure is most strongly associated with AMI. Especially traffic-related air pollution, including ultrafine exhaust particles, may be particularly related to AMI [30,31]. In Copenhagen there is very little contribution from traffic to PM_10 _urban background levels and this might be the reason for lack of associations in our study [32].

Adjustment for PM_10_, NO_2 _or CO in the cold season does not seem to have much effect on the temperature effect estimate at the different lags, except for CA5 (Figures 1 and 3). The fact that the temperature effect is robust to these adjustments suggests that air pollutants are not solely responsible for the higher risk during colder weather. Furthermore, the CA5 association is strengthened by adjustment for each air pollutant. It is possible that some of the previous studies may have missed the relationship between temperature and AMI due to lack of control for confounding by air pollution.

Advantages of our study include accurate meteorological, air pollution and health outcome data. Some disease misclassification is possible, but it is unlikely to be related to temperature. A study found that data from the Danish Hospital Registry and Danish Death Registry were valid for monitoring the population incidence of MI [33].

One limitation of the study is the assumption that the ambient air pollution levels, temperature and humidity measured in the inner city are the same across Copenhagen. The exposure error resulting from using ambient temperature and air pollution as a surrogate for personal exposure can potentially lead to bias in the estimated association, and this can be more pronounced among the elderly and other frail groups who generally spend most of their time indoors.

Another limitation of this study is the inability to adjust for PM_2.5_, as other studies observed strong associations with AMI [12].

In the GAM analysis, the population at risk must be very large relative to the daily number of events and the composition and size of the population at risk must not co-vary with the exposure of interest. The latter assumption may not be fully met whenever the susceptible portion of the total population at risk may be increased by the cumulative effects of prior exposures or decreased by the adverse effects of prior exposures (harvesting). The case-crossover design avoids both problems as the outcome is on an individual level and not a population level (daily number of events). Finally, similar results were in general observed for the association between Tapp_max _and AMI admissions in the case-crossover, GAM and GEE analyses.

Our results support the notion that moderate changes in ambient temperature are associated with AMI hospital admission. This association (assumed to be causal) is complex and depends on the specific health outcome (death or hospital admission), population characteristics (age, sex, SES), exposure conditions and the efficiency of the health care system, which all vary with time [34]. The International Panel on Climate Change stressed that many similar studies on temperature and health cannot be extrapolated infinitely into the future without considering major uncertainties regarding changes in populations, the rate and intensity of projected climate change and adaptation [35].

## Competing interests

The authors declare that they have no competing interests.

## Authors' contributions

JW and SL designed the study. MK and TE cleaned and contributed the air pollution and meteorological data. JW analysed the data. All authors contributed to writing and revising the manuscript and approve of the final manuscript.

## Financial support

The fee for the health outcome data extraction was partially funded by the Danish National Board of Health and the Danish Research Councils. The Danish Environmental Protection Agency funded the air pollution and meteorological measurements as part of the Danish Air Quality Monitoring Programme.

## Supplementary Material

Additional file 1 **Measurement Error in 24-hour Relative Humidity Measurements**. Click here for file
