# Supplementary material for: Apparent temperature and acute myocardial infarction hospital admissions in Copenhagen, Denmark: a case-crossover study
Source: Environ Health. 2012 Mar 30;11:19. doi: 10.1186/1476-069X-11-19 (PMC3353865; doi:10.1186/1476-069X-11-19)
Supplement: Additional file 1 — Measurement Error in 24-hour Relative Humidity Measurements. [file 1476-069X-11-19-S1.DOC]

**Supplementary Results**

**Measurement Error in 24-hour Relative Humidity Measurements**

The 24-hour relative humidity values decreased by about 10% during January 1999 to December 2006 (Supplementary Figure 1). This decrease is due to calibration problems and had a minor impact on the calculated apparent temperature over the range of relative humidity and temperature recorded in Copenhagen (Supplementary Figure 2, Supplementary Table 1). Hence, the error in the 24-hour relative humidity measurements is not likely to reduce the validity of the results.

**Supplementary Figure 1.** 24-hour average relative humidity measurements (lag0) in Copenhagen during 1 January 1999−31 December 2006.

**Supplementary Figure 2.** Theoretical relationship between apparent temperature, temperature and relative humidity.

**Supplementary Table 1 Meteorological conditions in Copenhagen during study period**

**(1 January 1999 − 31 December 2006).**

|  | **All year** | **Warm period** | **Cold period** |
| --- | --- | --- | --- |
| Number of days | 2922 | 1464 | 1458 |
| Relative humidity (%) |  |  |  |
| Number of days with missing data | 78 | 17 | 61 |
| Mean ± SD | 74 ± 11 | 69 ± 10 | 80 ± 9 |
| Minimum | 35 | 35 | 40 |
| Maximum | 100 | 99 | 100 |
| Percentiles |  |  |  |
| 25^th^ | 70 | 63 | 74 |
| 50^th^ | 75 | 70 | 81 |
| 75^th^ | 83 | 77 | 86 |
| Temperature (°C) |  |  |  |
| Number of days with missing data | 77 | 16 | 61 |
| Mean ± SD | 10 ± 7 | 15 ± 5 | 4 ± 4 |
| Minimum | -8 | 1 | -8 |
| Maximum | 25 | 25 | 16 |
| Percentiles |  |  |  |
| 25^th^ | 4 | 12 | 1 |
| 50^th^ | 10 | 15 | 4 |
| 75^th^ | 15 | 18 | 7 |

Warm period: April – September, Cold period: October – March. SD: Standard deviation
